# Intra- and inter-tumoural heterogeneity in von Hippel–Lindau disease-related renal cancer: a multimodal data study protocol

**DOI:** 10.1186/s41747-025-00648-0

**Published:** 2025-11-16

**Authors:** Isaline Rowe, Alberto Colombo, Francesca Corea, Francesco Pisu, Francesca Genova, Martina Uggé, Chiara Ciaparrone, Antonino Giangrasso, Giovanni B. Pipitone, Giulia M. Scotti, Alessandro Larcher, Giorgia Colciago, Marco J. Morelli, Roberta Lucianò, Paola Carrera, Pio Zeppa, Alessandro Caputo, Roberto Bertini, Francesco Montorsi, Andrea Salonia, Paolo Verze, Anna Palmisano, Antonio Esposito, Rosa Bernardi, Umberto Capitanio

**Affiliations:** 1 Comprehensive Cancer Center/Unit of Urology; URI; IRCCS Ospedale San Raffaele, Milan, Italy; 2 San Raffaele AI Center (S-RACE), Vita-Salute San Raffaele University, Milan, Italy; 3 Vita-Salute San Raffaele University, Milan, Italy; 4 Center for Omics Sciences, IRCCS Ospedale San Raffaele, Milan, Italy; 5 Comprehensive Cancer Center, IRCCS Ospedale San Raffaele, Milan, Italy; 6 Pathology Unit, AOU San Giovanni di Dio e Ruggi d’Aragona “Scuola Medica Salernitana”, Salerno, Italy; 7 Laboratory of Clinical Molecular Genetics, IRCCS Ospedale San Raffaele, Milan, Italy; 8 Department of Pathology, IRCCS Ospedale San Raffaele, Milan, Italy; 9 Genomics for Diagnosis of Human Disease Unit, IRCCS Ospedale San Raffaele, Milan, Italy; 10 Urology Unit, AOU San Giovanni di Dio e Ruggi d’Aragona “Scuola Medica Salernitana”, Salerno, Italy; 11 Advanced Imaging for Personalized Medicine Unit, Experimental Imaging Center, IRCCS Ospedale San Raffaele, Milan, Italy

**Keywords:** Carcinoma (renal cell), Magnetic resonance imaging, Multiomics, Organoids, von Hippel–Lindau disease

## Abstract

**Abstract:**

von Hippel–Lindau (VHL) disease is a rare hereditary cancer syndrome caused by germline pathogenic variants in the VHL gene. The current standard of care primarily involves surgical resection, which is arbitrarily recommended for renal tumours ≥ 3 cm to reduce the risk of metastasis. However, this approach often leads to repeated surgeries and increased patient morbidity. The key unmet clinical need for VHL patients is the ability to predict the most appropriate therapeutic strategy and the optimal timing for surgical intervention on an individualised basis. Here, we describe a methodology designed to create an integrated map of intra- and inter-tumour heterogeneity in VHL-associated clear cell renal cell carcinoma by combining radiomics, histology, RNA sequencing, whole genome sequencing, and patient-derived organoid cultures from multi-regional tumour biopsies. We hypothesise that decoding this heterogeneity through an integrated analysis of imaging, histopathology, and molecular profiling will enhance diagnostic accuracy and enable more informed and personalised therapeutic decisions for VHL patients.

**Relevance statement:**

Due to the current lack of biological or molecular markers assisting clinical decision-making, VHL patients undergo multiple surgical interventions with an incremental risk of complications and morbidity. We expect that our multimodal data study protocol will give tools to guide clinical management.

**Key Points:**

- Multiregional needle biopsies enable comprehensive analysis even in small ccRCC.
- Imaging characteristics suggest the presence of intra- and inter-lesion heterogeneity.
- Tumours are clonally independent and harbour distinct chromosome 3p loss events.
- Tumours display both intra- and inter-tumour transcriptomics heterogeneity.
- Patient-derived organoids grow more easily from areas of low tumour density.

**Graphical Abstract:**

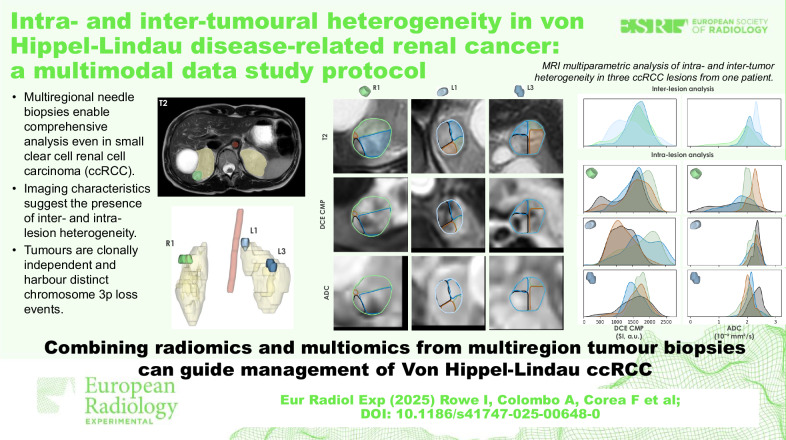

## Background

von Hippel–Lindau (VHL) disease is a hereditary syndrome affecting 1 in 36,000 people worldwide. It is caused by inherited pathogenic variants in the VHL gene, located on chromosome 3p25, whose protein product orchestrates cellular response to hypoxia [1, 2].

Patients develop multiple tumours in several organs, with clear cell renal cell carcinoma (ccRCC) being the primary cause of morbidity and mortality [3, 4]. The standard of care is surgical resection, arbitrarily recommended for tumours larger than three centimetres to minimise the risk of distant metastases [5]. Recently, a systemic hypoxia-inducible factor−HIF-2α inhibitor was approved by the Food and Drug Administration−FDA for adult patients with VHL-ccRCC not requiring immediate surgery [6–8]. Patients showed only partial responses, which may be caused by differences in drug efficacy across different tumours [6].

International guidelines recommend magnetic resonance imaging (MRI) screening every two years for patients affected with VHL aged between 15 and 65 years [9], based on its capability to characterise lesions in a multiparametric fashion and without radiation [10–12]. Recent research in VHL has explored the use of MRI features to phenotype ccRCC, predict growth, and assess grade [13–16], but its utility in assessing tumour heterogeneity remains unexplored. Furthermore, while genomic intra- and inter-tumour heterogeneity has been extensively documented in sporadic ccRCC [17–19], it remains poorly characterised in VHL-ccRCC. This gap leads to overtreatment of indolent lesions or underestimation of aggressive ones [20].

Here, we describe a comprehensive methodology to assess intra- and inter-tumour heterogeneity in small (< 4 cm) ccRCC, the most common type in VHL patients. The method integrates imaging, histological analysis, ribonucleic acid (RNA) sequencing, whole-genome sequencing (WGS), and patient-derived organoid (PDO) cultures from multiregional biopsies in multiple small tumours. This innovative approach could provide insights to guide clinical management in VHL-associated ccRCC.

## Materials and methods

### Ethics approval and consent to participate

All procedures were conducted after informed consent, adhering to the Declaration of Helsinki, and samples were stored at the CRB-OSR biobank (bbmri-eric:ID:IT_1383758011993577). The protocol PNRR-MR1-2022-12375818 received the approval of the Institutional Ethical Committee of IRCCS San Raffaele Hospital (Milan, approval date 14 December 2022), and informed consent was obtained from the patient included in the study. The trial was registered at ClinicalTrials.gov on March 7, 2024. The registration number is NCT06195150.

### Patients

**Fig. 1:**
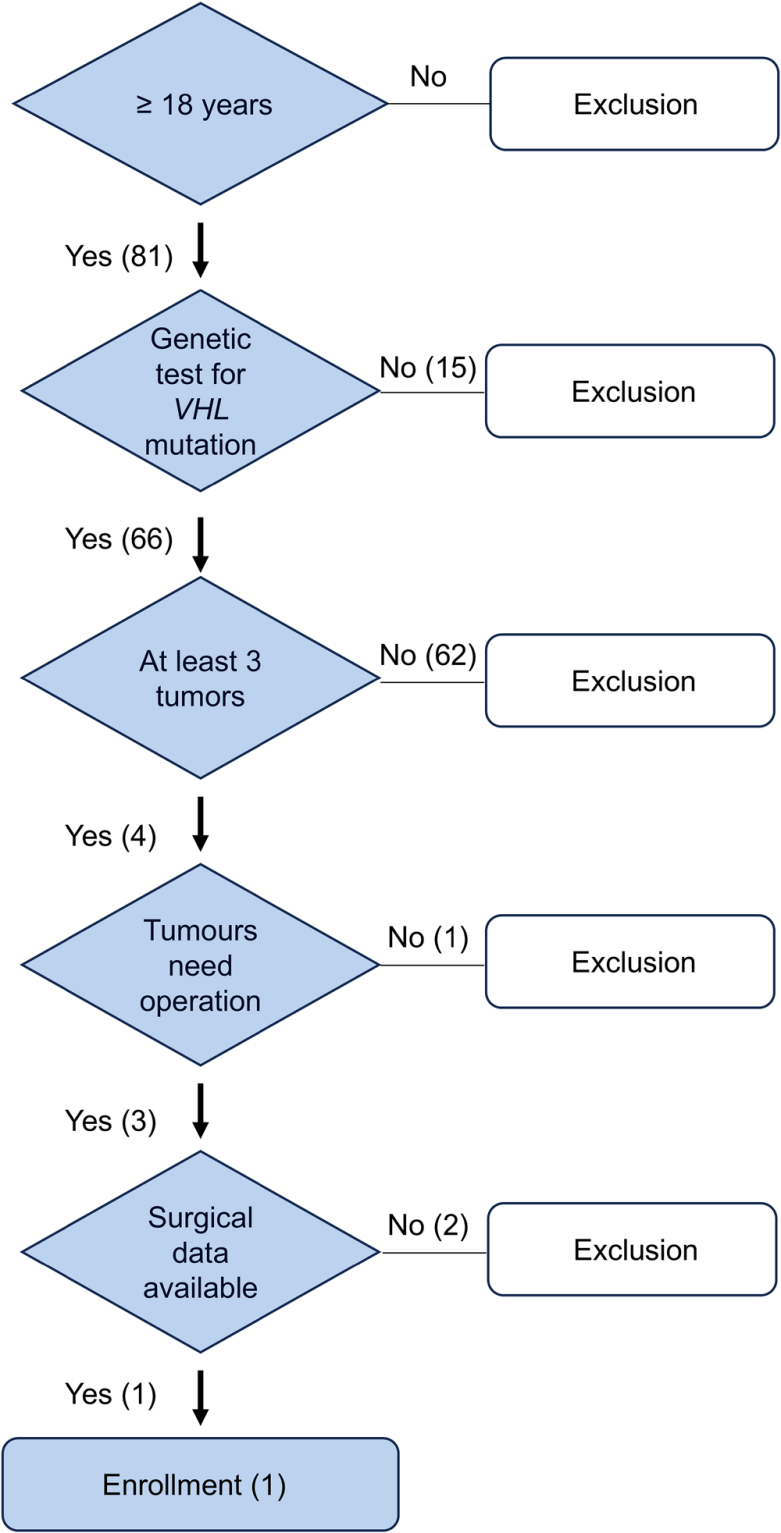
Flow chart of participants' inclusion criteria. Patient inclusion was based on the following criteria: age 18 or older, a confirmed genetic diagnosis of VHL disease, and the presence of at least three tumours requiring surgical intervention

The study involved a patient with a germline *VHL* mutation 563 T > C (pLeu188Pro). Participants' inclusion criteria are indicated in Fig. 1.

### MRI protocol

Presurgical examination was performed using a 1.5-T MR (Ingenia Ambition S, Philips Medical Systems, Best, the Netherlands) equipped with a 32-channel phased-array coil one week before surgery. The acquisition protocol (details in Supplementary Table S1) included T2-weighted (T2W), dual-echo T1-weighted, and diffusion-weighted image (DWI) sequences (used to compute the apparent diffusion coefficient (ADC) map), as well as a multiphase dynamic contrast enhancement (DCE) study, to obtain morphological, fat content, cellularity, and vascularisation characterisation, respectively.

### Image processing

**Fig. 2:**
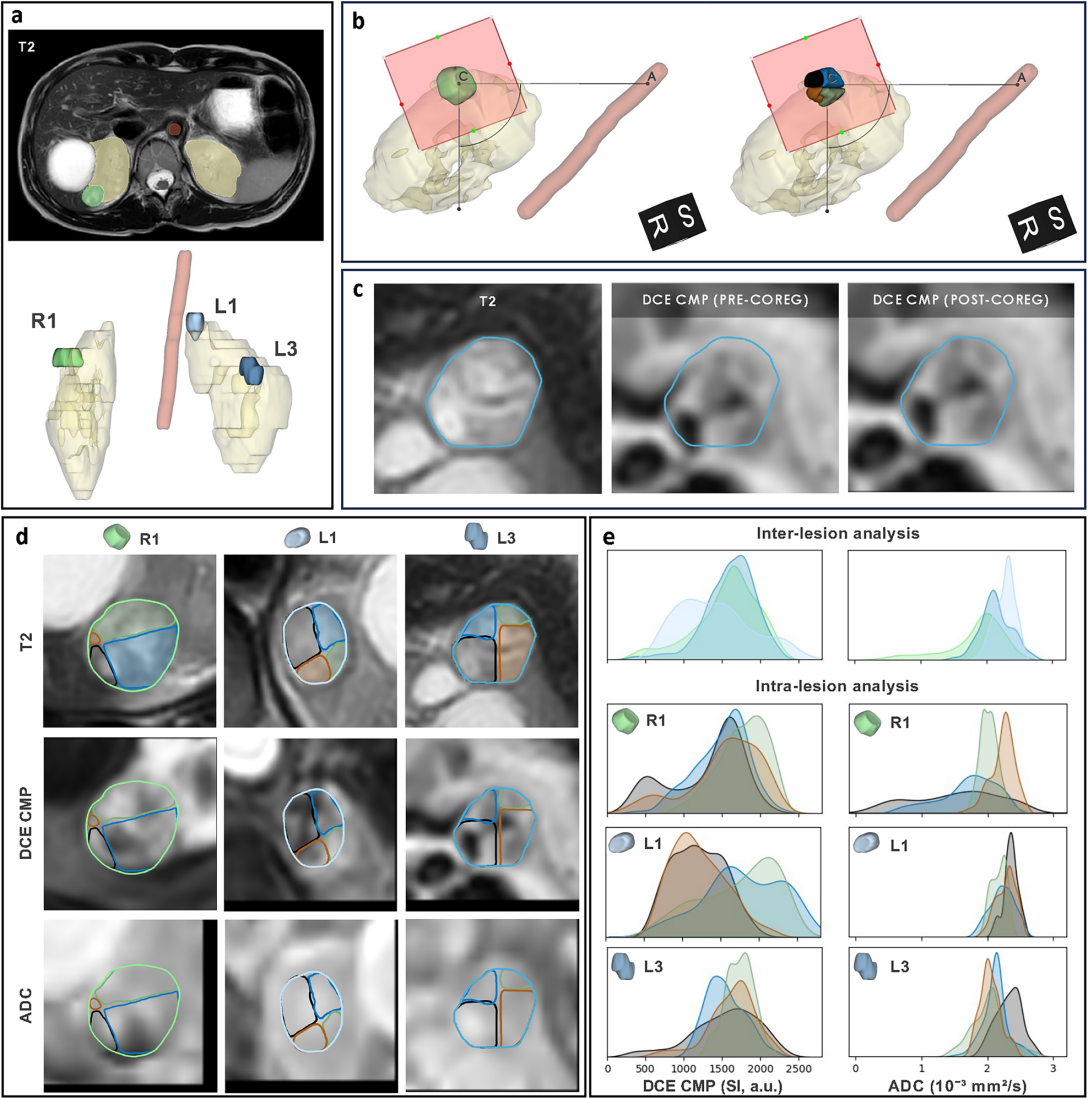
Postprocessing and multiparametric analysis of magnetic resonance images revealing intra- and inter-tumour heterogeneity. Postprocessing: (**a**) segmentation of left and right kidneys, lesions (R1, L1, and L3), and the descending aorta using T2W images; (**b**) definition of a lesion-specific reference system based on identifying the approximated resection plane (red square) and the axis connecting the lesion centre C to the descending aorta A, enabling the subdivision of each lesion into four quadrants; (**c**) lesion-specific multi-parametric three-dimensional patches, *e.g*., DCE corticomedullary phase (DCE CMP) of L3 lesion shown in the panel) are extracted and coregistered with the T2W patches. Multiparametric analysis: (**d**) whole-lesion and quadrant-specific contours are highlighted on axial views of T2W images, DCE CMP images, and ADC maps. **e** Kernel density estimate plots compare DCE CMP, and ADC value distributions between whole lesions R1, L1, and L3 (inter-lesion analysis) and between quadrants within each lesion (intra-lesion analysis)

Tumours, kidneys, and descending aorta were manually outlined on T2W images (Fig. 2a) by a radiologist with 15 years of experience in abdominal imaging (AP) using 3D Slicer (https://slicer.org). Lesions were labelled by kidney side (R = right, L = left). A standardised procedure ensured consistent analysis across imaging and excised tumour specimens: first, a lesion-specific reference system was defined, based on identifying the lesion resection plane and the axis (CA) connecting the centre of the mass (C) to the descending aorta (A). Then, each lesion was divided into four quadrants using two cutting planes perpendicular to the resection plane and passing through the centre of the mass, with the former being parallel and the latter perpendicular to the axis CA. Finally, each quadrant of each lesion was colour-coded as black, blue, green, or brown, matching surgical specimen staining (Fig. 2b).

Preprocessing for multiparametric analysis included resampling to ensure uniform geometry across the contrasts, signal intensity quantisation (100 levels), extraction of three-dimensional patches centred in each tumour’s centre of mass and their coregistration. Coregistration of patches extracted from DWI with a *b*-value of 800 s/mm² (b800), ADC map, and each phase of the DCE study to patches extracted from T2W images, used an affine transformation and mutual information as metric (Fig. 2c).

In addition, radiomic feature maps were extracted from whole lesion masks applied to T2W images using PyRadiomics v 3.1.0 21 [21] (extraction parameters summarised in Supplementary Table S2). Prior to feature extraction, the original T2W images were resampled to a voxel spacing of 1 × 1 mm on the axial plane, while the slice thickness remained unchanged. Image interpolation was performed using a B-spline method, whereas the masks were interpolated using the nearest neighbour approach. A fixed bin width of 25 was applied for intensity discretisation. Features were extracted on a voxel basis and ensuring extraction occurred within the axial plane only. A total of 51 radiomic feature maps were computed, including first-order statistics and texture features (grey level size zone matrix, neighbouring grey tone difference matrix, and grey level dependence matrix).

As part of the feature selection process, radiomic features exhibiting high redundancy, defined as a Spearman’s correlation coefficient (ρ) greater than 0.6 with either T2W image intensity or other radiomic features, were excluded. From the subset of nonredundant features, one first-order and one texture feature were selected based on the highest effect sizes observed at both intra-tumoural and inter-tumoural levels.

Finally, for each lesion considered as a whole, as well as for each quadrant of each lesion, voxel count, volume, and signal distribution (mean ± standard deviation) were calculated.

### Sampling of multiregional biopsies

The excised tumour was divided into four quadrants, and 16-G needle biopsies were taken from each region.

### Histology of multiregional biopsies and surgical sections

Histological evaluation was performed by pathologists with dedicated experience in the diagnosis of RCC. Biopsies and surgical sections were stained with haematoxylin and eosin (Ventana HE 600 system, Roche) and assessed for World Health Organization/International Society of Urological Pathology (WHO/ISUP) grade (from 1 to 4), stage, necrosis, angioinvasion, and tumour cell percentage.

### Simultaneous purification of DNA and RNA

Deoxyribonucleic acid (DNA) and RNA were copurified using the AllPrep DNA/RNA Micro Kit (QIAGEN). Integrity was assessed with TapeStation, and DNA quantified with Qubit. Full protocol in Supplementary Material.

### RNA sequencing and transcriptomic data analysis

RNA-seq libraries were prepared using the “Low_Input_mRNA_Novaseq6000_Gb” protocol and sequenced as 100 nt single-end reads on the NovaSeq 6000 platform (Illumina) (details in Supplementary material). To assess intertumour transcriptomics heterogeneity, we compared each tumour to the others, considering each region as a replicate for its respective tumour. Principal component analysis (PCA) was performed for the three pairwise comparisons. A separate PCA analysis was performed to evaluate global gene expression variation across tumour quadrants and for a correlation analysis with imaging features. For the two PCs explaining more variance, loadings were used to identify the top 50 contributing genes, which were used for functional enrichment analysis.

### WGS

DNA libraries were prepared using a polymerase chain reaction-free protocol. Sequencing was performed on the NovaSeq6000 platform (150 base pairs paired-end). Reads were aligned to hg38 (Alt-Masked v3) using Dynamic Read Analysis for GENomics (DRAGEN); somatic variants were identified with the DRAGEN Somatic pipeline (v4.2.7, tumour-normal mode), and germline variants from blood using DRAGEN Germline (v4.2.4). Further details are provided in the Supplementary material.

### PDOs

Tumour biopsies were dissociated using the Miltenyi Tumour Dissociation Kit and gentleMACS™ system, filtered, and treated with erythrocyte lysis buffer. Cells were embedded in 75% Matrigel and cultured in supplemented DMEM/F-12 (details in Supplementary material).

### Statistical analysis

Associations between imaging and molecular variables, which included histologic, transcriptomic, and genomic features, were assessed using Spearman’s rank correlation coefficient (ρ). For imaging data, normality of voxel value distributions was assessed using the Shapiro–Wilk test, and a nested ANOVA was applied to compare groups, quantifying significance and effect size, followed by pairwise *post hoc t*-tests with Bonferroni-adjusted *p*-values [22]. Values of *p* < 0.05 were deemed statistically significant. Analyses were performed using the Python language (version 3.9).

## Results

### Image postprocessing of three ccRCC lesions from one VHL patient

We developed a method to analyse three lesions of a female—29-years-old patient with VHL syndrome and multiple renal lesions, of both cystic and solid appearance (Fig. 2d). The patient has been a candidate for surgical excision according to the number, localisation and kinetics of the lesions according to established expert-based recommendations [23].

The three segmented lesions R1, L1, and L3, had volumes of 3.7 cm³, 2.3 cm³, and 2.9 cm³, respectively. Inter-tumour heterogeneity was observed at imaging (Fig. 2e and Table 1). Lesion L1, compared to R1 and L3, showed lower signal intensity values on DWI b800 images (204.6 ± 19.6 *versus* 239.5 ± 48.6 and 215.6 ± 41.1, respectively, *p* < 0.001), higher values on ADC images (2.3 ± 0.2 *versus* 1.8 ± 0.5 and 2.1 ± 0.2 × 10⁻³ mm²/s, respectively, *p* < 0.001) (Supplementary Table S3). Consistently, the contrast enhancement of L1 was lower compared to the other lesions on perfusion images, suggesting a higher extent of intralesional fluid content (Fig. 2e and Supplementary Table S3). T2W signal intensity was significantly lower in R1 compared to L1 and L3 (*p* < 0.001). In the radiomic analysis of the six non-correlated features, L3 had a significantly higher FirstOrder_InterquartileRange than R1 and L1 (*p* < 0.001), and GLSZM_GrayLevelNonUniformity was greater in R1 compared to both L3 and L1 (*p* = 0.002–0.009). Comparing individual quadrants within the same lesion revealed intralesional heterogeneity in lesions R1, L1 and L3 (Fig. 2e, Table 1, and Supplementary Table S4). The Black quadrant of R1 lesion (R1 Black) and R1 Blue showed lower ADC values compared to R1 Green and R1 Brown (1.5 ± 0.7 and 1.7 ± 0.4 × 10⁻³ mm²/s *versus* 2.0 ± 0.2 and 2.2 ± 0.2 × 10⁻³ mm²/s, respectively, *p* < 0.001), consistent with higher cellularity. Conversely, L3 Black exhibited reduced DWI b800 signal intensity (182.4 ± 38.7 *versus* ≥ 219.3 ± 22.8, *p* < 0.001) and elevated ADC values (2.3 ± 0.2 *versus* ≤ 2.1 ± 0.2 × 10⁻³ mm²/s, *p* < 0.001), compared to the other quadrants of the same lesion. In L1, T2W intensity varied significantly between quadrants (*p* < 0.001), with the Black quadrant showing higher values than Green and Blue. In L3, the FirstOrder_InterquartileRange was also significantly higher in the Brown quadrant compared to Green and Blue (*p* < 0.001).

**Table 1:** Histological, WGS and imaging data were obtained in each biopsy

Tumours	L1				L3				R1			
Area	Black	Blue	Green	Brown	Black	Blue	Green	Brown	Black	Blue	Green	Brown
Kidney	Left	Left	Left	Left	Left	Left	Left	Left	Right	Right	Right	Right
Histology	ccRCC	ccRCC	ccRCC	ccRCC	ccRCC	ccRCC	ccRCC	ccRCC	ccRCC	ccRCC	ccRCC	ccRCC
Stage	pT1a pNX	pT1a pNX	pT1a pNX	pT1a pNX	pT1a pNX	pT1a pNX	pT1a pNX	pT1a pNX	pT1a pNX	pT1a pNX	pT1a pNX	pT1a pNX
Fuhrman grade	G1/G2	G1/G2	G1/G2	G1/G2	G1/G2	G1/G2	G1/G2	G1/G2	G1/G2	G1/G2	G1/G2	G1/G2
Max diameter (cm)	1.2	1.2	1.2	1.2	1.2	1.2	1.2	1.2	1.7	1.7	1.7	1.7
Necrosis	NO	NO	NO	NO	NO	NO	NO	NO	NO	NO	NO	NO
Angioinvasion	NO	NO	NO	NO	NO	NO	NO	NO	NO	NO	NO	NO
% tumour cells (specimen)	< 10%	10%	25%	15%	30%	40%	50%	35%	70%	20%	15%	50%
% tumour cells (biopsies)	NA	10%	5%	5%	NA	50%	50%	30%	50%	50%	40%	NA
Tumour purity (DRAGEN pipeline)	NA	30%	NA	NA	43%	54%	47%	NA	62%	65%	58%	61%
Chr3p loss	NA	YES	NA	NA	YES	YES	YES	NA	YES	YES	YES	YES
Chr3 breakpoint	NA	p12.3	NA	NA	q13.11	q13.11	q13.11	NA	q12.1	q12.1	q12.1	q12.1
PDO growth	YES	YES	YES	YES	NO	NO	NO	NO	NO	NO	NO	NO
PDO Estimated tumour purity (DRAGEN)	58%	NA	NA	39%								
PDO Chr3p loss	NO	NA	NO	NO								
DWI b800 (SI a.u.)	199.2 ± 12.2	207.4 ± 26.6	199.5 ± 25.9	211.4 ± 13.6	182.4 ± 38.7	219.3 ± 22.8	243.4 ± 30.8	228.1 ± 35.9	232.1 ± 53.4	259.4 ± 53.4	233.8 ± 30.1	201.4 ± 36.7
ADC (10⁻³ mm²/s)	2.4 ± 0.1	2.2 ± 0.2	2.2 ± 0.1	2.3 ± 0.1	2.3 ± 0.2	2.1 ± 0.2	2.0 ± 0.3	2.0 ± 0.2	1.5 ± 0.6	1.7 ± 0.4	2.0 ± 0.1	2.2 ± 0.2
DCE PRE (SI a.u.)	409.8 ± 54.6	408 ± 72	501.5 ± 46.7	451.3 ± 39.1	599.2 ± 143.6	498.9 ± 87.5	537.7 ± 107.4	635.4 ± 177.8	414.8 ± 108.8	427.3 ± 47.2	432.4 ± 38.7	424.9 ± 69.8
DCE CMP (SI a.u.)	1,207.6 ± 337.2	1,785.4 ± 511.6	1,725.1 ± 499.6	1,214.1 ± 353.7	1,533.6 ± 438.6	1,541.6 ± 251.5	1,722.7 ± 186.4	1,622.1 ± 318.4	1,292.7 ± 480.2	1,487.5 ± 353.6	1,763.3 ± 315.8	1,531.5 ± 466.5
DCE NP (SI a.u.)	1,571.5 ± 431.2	2,001.5 ± 560	1,997.6 ± 549.4	1,564.9 ± 455.6	1,929 ± 558.6	1,782.2 ± 329.4	1,878.6 ± 234.4	2,020 ± 289.5	1,695.7 ± 542.4	1,779.5 ± 350.5	1,970.4 ± 273.9	1,806.9 ± 620.5
DCE EP (SI a.u.)	1,908 ± 460.4	2,242.2 ± 472.3	2,100.2 ± 469.6	1,768.9 ± 439.9	1,988.7 ± 554.9	1,729.6 ± 387.8	1,907.8 ± 226.2	2,140.5 ± 336	1,739.9 ± 611.5	1,837.5 ± 422.2	2,045.5 ± 316.8	1,897.3 ± 663.2
T2W, mean (SI a.u.)	1,658.9 ± 187.5	1,451 ± 207.4	1,380.0 ± 175.0	1,622.7 ± 172.4	1,587.2 ± 293.2	1,589.1 ± 203.8	1,422.2 ± 164.4	1,448.4 ± 273.0	1,342.1 ± 246.8	1,310.1 ± 195.8	1,254.7 ± 200.4	1,303.2 ± 249.0
FirstOrder_–_Interquartilerange (SI a.u.)	133.2 ± 72.2	151.6 ± 83.9	154.7 ± 76.5	119.7 ± 64.8	186.9 ± 105.6	155.5 ± 122.7	129.4 ± 76.0	235.5 ± 141.9	139.5 ± 118.3	125.5 ± 71.5	126.7 ± 58.9	160.5 ± 108.5
GLSZM_–_GrayLevelNonUniformity	1.1 ± 0.2	1.1 ± 0.1	1.1 ± 0.2	1.1 ± 0.2	1.1 ± 0.2	1.1 ± 0.2	1.1 ± 0.2	1.1 ± 0.1	1.2 ± 0.2	1.1 ± 0.2	1.1 ± 0.2	1.1 ± 0.2

L1 Black, L3 Black and R1 Brown, had insufficient and deteriorated diagnostic material, preventing histological identification. DNA extracted from L3 Brown has poor DNA quality, so it was excluded from whole-genome sequencing. Empty cells: No samples were available for analysis due to the absence of PDO growth

*ADC* Apparent diffusion coefficient, *CcRCC* Clear cell renal cell carcinoma*, Chr3* Chromosome 3*, CMP* Corticomedullary phase, *DCE* Dynamic contrast enhancement, *DWI* Diffusion-weighted imaging, *DRAGEN* Dynamic read analysis for GENomics, *EP* Excretory phase, *G1/G2* Grade 1/grade 2, *GLSZM* Grey level size zone matrix, *NA* Not available, *NP* Nephrographic phase, *PDO* Patient-derived organoids*, PRE* Precontrast phase, *SI a.u*. Signal intensity, arbitrary units, *T2W* T2-weighted

### Histology of multiregional biopsies and surgical sections

Histological assessment of multiregional biopsies revealed ccRCC, staged as pT1a pNX, and graded as grade 1 (WHO/ISUP). No necrosis or angioinvasion was observed. Tumour cellularity ranged from 5% to 10% in L1, 30% to 50% in L3, and 40% to 50% in R1 lesions (Table 1).

Histopathological evaluation of corresponding surgical sections confirmed ccRCC, pT1a pNX stage, G2 grade, and the absence of necrosis or angioinvasion. The percentage of neoplastic tissue ranged from 10% to 25% in L1 lesions, 30% to 50% in L3 lesions, and 15% to 70% in R1 lesions (Table 1 and Supplementary Fig. S1).

### Transcriptomic analysis of multiregional biopsies

**Fig. 3:**
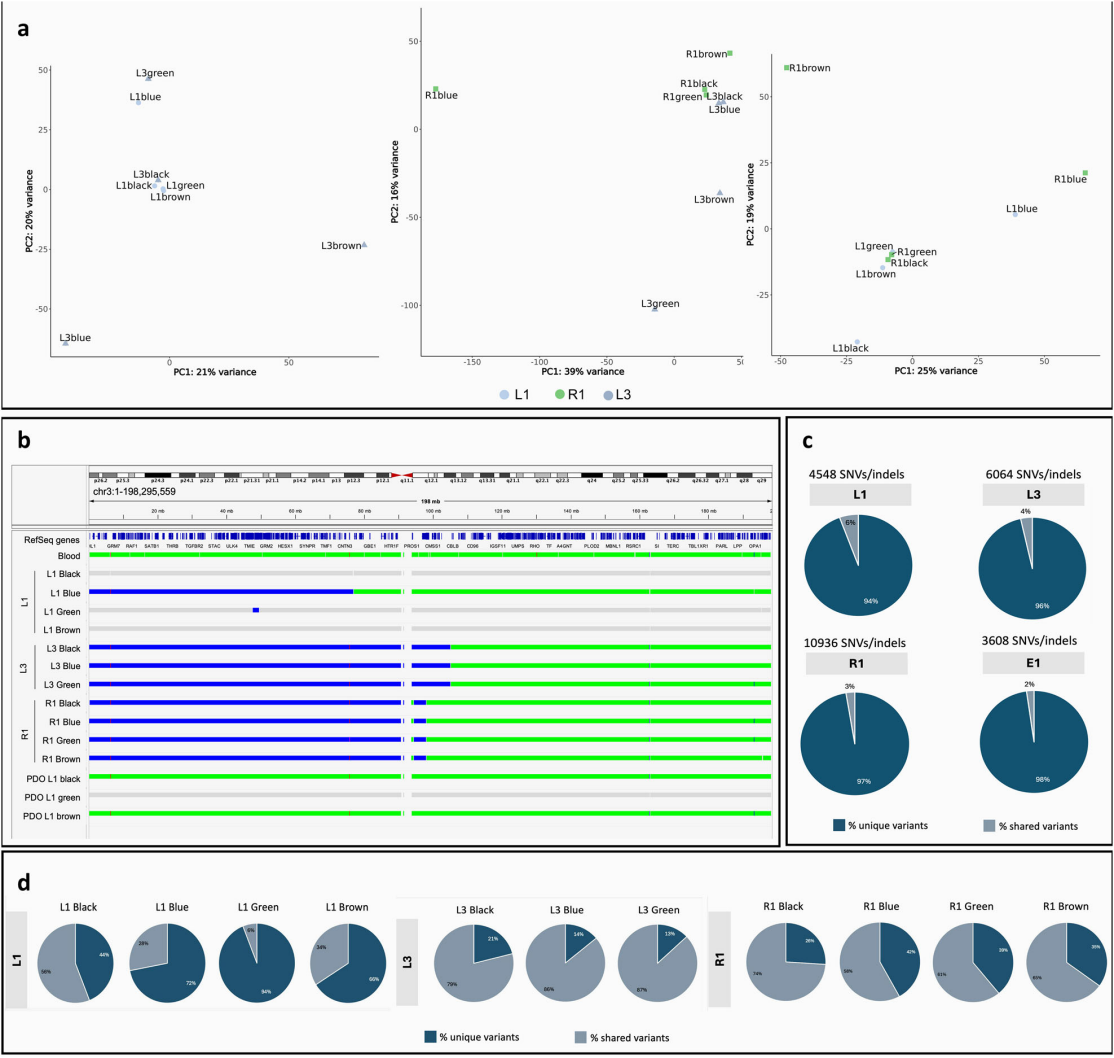
Multiregional biopsy RNA sequencing and WGS. **a** PCA of the multiregion samples of L1 *versus* L3, R1 *versus* L3, and R1 *versus* L1. **b** Integrative genomics viewer (IGV) visualisation of copy number status of chromosome 3 across the different tumour lesions and the germline sample. The events are colour-coded according to the type of CNV: wild-type copy number status in green, deletions (copy number = 1) in blue, and light grey for events not passing the quality filters. **c** Inter-tumour heterogeneity analysis. Pie charts show the percentages of unique (dark coloured) or shared (light coloured) SNVs/indels across all tumours. The total number of high-confidence SNVs/indels is indicated for each tumour. **d** Intra-tumour heterogeneity analysis. Pie charts show the percentages of unique (dark coloured) or shared (light coloured) SNVs/indels across all regions within each tumour. Indels, Insertions or deletions; SNVs, Single nucleotide variants

To evaluate intertumour transcriptomics heterogeneity, we compared each tumour to the others, treating each area as a replicate for its respective tumour. PCA, performed for the three pairwise comparisons (L1 *versus* L3, R1 *versus* L3, and R1 *versus* L1), showed the samples did not cluster according to their tumour identity (Fig. 3a), indicating that intratumour heterogeneity was as pronounced as intertumour heterogeneity. This finding was further supported by differential gene expression analysis, which did not identify significant differences in genes across the three comparisons.

At the distinct PCA performed to evaluate gene expression variation across all the tumour quadrants, the first two PCs, PC1 and PC2, accounted for the highest proportion of variance in the transcriptomic data, with PC1 explaining 14.5% and PC2 12.1% of the total variance.

### WGS analysis from multiregional biopsies

We sequenced the whole genomes of 11 tumour biopsies, excluding L3 Brown due to poor DNA quality. Tumour purity, estimated by the DRAGEN algorithm, exceeded the 30% threshold for all samples except three biopsies from the L1 tumour (Black, Green, and Brown), where purity was insufficient for algorithmic estimation (Table 1 and Supplementary Fig. S2). 3p loss, resulting in VHL loss of heterozygosity, was observed in all tumours except these three. The 3p breakpoint mapped to three distinct genomic locations (Fig. 3b), suggesting clonal independence among the tumours. The intertumour single-nucleotide variants analysis showed over 95% unique variants in each tumour, underscoring their independent clonal origins and high intertumour heterogeneity (Fig. 3c). Comparing high-confidence single-nucleotide variants across different regions within each tumour, L3 and R1 tumours displayed limited intratumour heterogeneity, with shared variants predominating over unique ones (Fig. 3d), suggesting minimal subclonal populations within these tumours.

### PDOs generation

PDOs were successfully generated from 33.3% of the analysed biopsies, albeit few organoids were generated/biopsy, except for the biopsies from the L1 tumour (Table 1 and Supplementary Fig. S3). Notably, these PDO cultures originated specifically from biopsies that exhibited low tumour density (Table 1). We extracted DNA from all four areas of PDO L1, but we succeeded in performing WGS only with three of them (*i.e*., L1 Black, L1 Green, and L1 Brown). PDO L1 Blue concentration was too low to sequence, so we excluded it. This is in line with the fact that the organoid coming from the tumour area with the relatively highest tumour purity is the most difficult to grow in culture. Consistently, WGS data showed normal 3p copy number (Fig. 3b), suggesting that these organoids are not composed of tumour cells.

### Correlation analysis between imaging and molecular features

The correlation analysis results between imaging and molecular features are illustrated in Supplementary Fig. S4. Regarding histologic features, as already well established [24], the estimated percentage of tumour cells in the biopsies showed a significant strong positive correlation with DWI b800 (ρ = 0.78, *p* = 0.014) and a significant strong negative correlation with ADC (ρ = -0.79, *p* = 0.011), while the percentage of tumour cells in the clinical specimens were moderately negatively correlated with ADC (*ρ* = -0.52, *p* = 0.084), implying an association between restricted molecular water diffusivity (high DWI b800 signal intensity and low ADC) and the percentage of tumour cells observed at histology.

Considering the PCs explaining the highest proportion of variance in the transcriptomic data across all the tumour quadrants, PC1 showed a negative correlation with DWI b800 (ρ = -0.48, *p* = 0.112) and a significant positive correlation with FirstOrder_InterquartileRange (ρ = 0.64, *p* = 0.026), suggesting that this component captures a transcriptomic signal associated with lower cellular density (low DWI b800) and higher intralesional heterogeneity on T2W images (high FirstOrder_InterquartileRange). The enrichment of pathways explaining these correlations includes lysosomal and intracellular acidification, proteolysis, apoptosis, Organic Anion Transporters, and Glutamate and Glutamine Metabolism (Supplementary Fig. S5). PC2 exhibited the opposite trend, being positively correlated with DWI b800 and negatively correlated with ADC (*ρ* = 0.49, *p* = 0.106 and ρ = -0.42, *p* = 0.179), implying an association with higher cellularity and restricted diffusion. This can be explained by the enrichment pathways indicating enhanced cellular proliferation, active gene expression and translation, epithelial-to-mesenchymal transition, angiogenesis, hypoxic response, immune activation, and altered amino acid metabolism (Supplementary Fig. S5).

When examining genomic features, tumour purity estimated by the DRAGEN algorithm showed a significantly strong negative correlation with ADC (ρ = -0.71, *p* = 0.048) and, notably, a significantly strong negative correlation with DCE cortico-medullary and nephrogenic phase enhancement (ρ = -0.76, p = 0.028 and ρ = -0.79, p = 0.021, respectively). These findings may reflect the complex and heterogeneous nature of VHL-associated ccRCC. Regions with high tumour purity, dominated by densely packed malignant clear cells, likely restrict water diffusion, resulting in low ADC values. Areas with lower tumour purity—containing heterogeneous cell types (tumour cells, stromal cells, and endothelial tissue cells)—may exhibit higher perfusion (high cortico-meadullary phase DCE) and increased vascular permeability and extracellular volume (high nephrogenic phase DCE).

## Discussion

This proof-of-concept study and protocol presentation explores the potential feasibility of a combined imaging and multiregion biopsies approach for characterising intra- and inter-tumour heterogeneity in small VHL disease-associated ccRCCs. We find that multiregional needle biopsies in small ccRCC reveal intra- and intertumour heterogeneity in imaging and transcriptomics. Despite universal VHL loss of heterozygosity, tumours are clonally independent. Notably, PDOs are more successfully established in regions with lower tumour cell density. Imaging characteristics analyses suggest the presence of intra- and inter-lesion heterogeneity and show significant correlations with the considered histologic, transcriptomic and genomic features.

In tumours characterised by a high degree of heterogeneity with necrosis or cystic components, such as ccRCC, the assessment of whole-lesion MRI quantitative parameters may lead to underestimating tissue density and vascularisation, resulting in an underestimation of cellularity and aggression in the cancer [25]. Therefore, the ability to noninvasively map focal tumour regions characterised by ADC and perfusion alterations associated with increased cellularity at histology may improve clinical management by potentially enabling MRI-guided biopsies targeting the most solid and viable tumour areas. Also, this would impact treatment selection and risk stratification.

Furthermore, our findings suggest that ADC, DWI and heterogeneity of T2W signal intensity are linked not only to structural tumour properties but also to complex molecular programs characteristic of metabolically active tumours. In particular, the correlations with the PCs derived from transcriptomic data, suggest a link to distinct transcriptomic programs in ccRCC, highlighting underlying differences in tumour biology such as down regulated pathways in ccRCC such as impaired lysosomal function, and up regulated pathways in ccRCC apoptosis (PC1), *versus* proliferative, hypoxic, and immunologically active profiles (PC2), and may offer complementary insights for tumour characterisation beyond histopathology [26].

Genomic analyses confirmed *VHL* loss of heterozygosity in all tumours resulting from the combination of the germline variant and somatic 3p loss in line with previous observations [27]. The distinct 3p breakpoints and the predominance of unique single-nucleotide variants (insertions or deletions) in each tumour indicate independent clonal origins. Multiregional sampling within the same tumour does not identify significant subclonal populations, suggesting relative intra-tumour genomic homogeneity consistent with previous findings [28, 29]. Instead, we observe both intra- and inter-tumour heterogeneity at the whole transcriptome level. In our study, we faced challenges in generating PDOs that accurately represented ccRCC tumour cells from low-grade tumour biopsies (Grade 1/Grade 2 Fuhrman grade [20]). While viable cultures were derived from 33.3% of the biopsies, the proportion of cancer cells was very low and not representative of the original tumours. These findings highlight the difficulty of faithfully capturing the ccRCC tumour cell population in PDO models, particularly with low-grade tumours.

This study establishes a proof-of-concept framework for assessing intra- and inter-tumoural heterogeneity in a VHL disease-associated ccRCC. However, while they are strong enough to show the feasibility of the methodology, we are aware that caution is required in interpreting statistical significances. The findings require validation in larger cohorts, including both VHL disease-related and sporadic ccRCC cases, to improve their robustness and further validate their applicability. The data of intra- and inter-tumour heterogeneity at the level of transcriptomics and tumour density, highlight the importance of sampling multiple regions from different tumours for an accurate assessment of tumour biology. It gives a more accurate picture of the molecular characteristics that are crucial to guide management decisions.

It is possible that the application of this protocol would lead to potential aspects, such as the risk of performing multiple biopsies on a single patient. Moreover, in subsequent projects, it will be necessary to analyse the cost-effectiveness of such a protocol across countries to better define the applicability of the entire process.

In conclusion, the combined imaging and multi-region biopsy approach has the potential to improve the clinical management of VHL disease-associated ccRCCs by identifying unique imaging, cellular, and molecular characteristics within each tumour. A tailored characterisation of VHL disease-associated ccRCC can avoid under- and overtreatments, which currently severely affect the management of these patients.

## Supplementary information

**Additional file 1: Fig. S1.** Histology assessment of biopsies and surgical specimen and patient-derived organoid generation. Representative example of histological evaluation of one of the multi-regional biopsies and their corresponding surgical specimens. (**a**) L1 Blue biopsy (upper panel) and corresponding surgical section (lower panel); (**b**) L3 Blue biopsy (upper panel) and corresponding surgical section (lower panel); (**c**) R1 Black biopsy (upper panel) and corresponding surgical section (lower panel). **Fig. S2.** Quality control of RNA and DNA sequencing. Quality control (QC) of RNA sequence: (**a**) percentage of assignment, mapping and alignment; (**b**) millions of assignments and alignment; (**c**) read distribution in each sample. (d) Mapping metrics. Total number of input reads and mapped reads for each sample. (e) The average coverage over genome was above the recommended target for short-read whole genome sequencing using Illumina platforms (≥ 60× tumour and ≥ 30× blood). (**f**) Estimated tumour purity based on Dynamic Read Analysis for GENomics−DRAGEN somatic pipeline output. NA Not available (assigned to samples where the algorithm could not estimate a final tumour model). **Fig. S3.** Patient-derived organoids (PDOs) generation. (**a**-**d**) PDOs cultures from four different tumour regions: (a) from tumour L1 black region; (**b**) from tumour L1, blue region; (**c**) from tumour L1, green region; (**d**) from tumour L1, brown region. **Fig. S4.** Spearman correlation between imaging and histologic, transcriptomics and genomic continuous variables. **Fig. S5.** Enriched pathways in PC1 and PC2. (**a**) Principal component 1 (PC1) was found to be significantly associated with the enrichment of pathways involved in lysosomal and intracellular acidification, proteolysis, apoptosis, organic anion transporters, and glutamate and glutamine metabolism. (**b**) Principal component 2 (PC2) was found to be significantly associated with the enrichment of pathways involved in involved in cellular proliferation, active gene expression and translation, epithelial-to-mesenchymal transition (EMT), angiogenesis, hypoxia response, immune activation, and aminoacid metabolism. **Table S1.** Magnetic resonance imaging acquisition parameters. **Table S2.** Radiomics features maps extraction parameters. **Table S3.** Nested ANOVA results – Multiparametric analysis. **Table S4** Nested ANOVA results – Radiomics.

## Data Availability

The RNA-seq data have been deposited in the Gene Expression Omnibus repository under the series record GSE288008. A secure token has been generated to enable the review of record GSE288008 while it remains in private status; the token is available upon request. The WGS sequencing data have been deposited at the European Genome-phenome Archive (EGA), which is hosted by the EBI and the CRG, under accession number EGAS50000000295.

## Abbreviations

ADC: Apparent diffusion coefficient
b800: 800 s/mm²
CcRCC: Clear cell renal cell carcinomas
DCE: Dynamic contrast enhancement
DNA: Deoxyribonucleic acid
DRAGEN: Dynamic read analysis for genomics
DWI: Diffusion-weighted images
MRI: Magnetic resonance imaging
PC: Principal component
PCA: Principal component analysis
PDO: Patient-derived organoid
RNA: Ribonucleic acid
T2W: T2-weighted
VHL: von Hippel–Lindau
WGS: Whole-genome sequencing
WHO/ISUP: World Health Organization/International Society of Urological Pathology
