# Supplementary material for: Intra- and inter-tumoural heterogeneity in von Hippel–Lindau disease-related renal cancer: a multimodal data study protocol
Source: Eur Radiol Exp. 2025 Nov 16;9:114. doi: 10.1186/s41747-025-00648-0 (PMC12620338; doi:10.1186/s41747-025-00648-0)
Supplement: Supplementary file 1 — Additional file 1: Fig. S1. Histology assessment of biopsies and surgical specimen and patient-derived organoid generation. Representative example of histological evaluation of one of the multi-regional biopsies and their corresponding surgical specimens. (a) L1 Blue biopsy (upper panel) and corresponding surgical section (lower panel); (b) L3 Blue biopsy (upper panel) and corresponding surgical section (lower panel); (c) R1 Black biopsy (upper panel) and corresponding surgical section (lower panel). Fig. S2. Quality control of RNA and DNA sequencing. Quality control (QC) of RNA sequence: (a) percentage of assignment, mapping and alignment; (b) millions of assignments and alignment; (c) read distribution in each sample. (d) Mapping metrics. Total number of input reads and mapped reads for each sample. (e) The average coverage over genome was above the recommended target for short-read whole genome sequencing using Illumina platforms (≥ 60× tumour and ≥ 30× blood). (f) Estimated tumour purity based on Dynamic Read Analysis for GENomics−DRAGEN somatic pipeline output. NA Not available (assigned to samples where the algorithm could not estimate a final tumour model). Fig. S3. Patient-derived organoids (PDOs) generation. (a-d) PDOs cultures from four different tumour regions: (a) from tumour L1 black region; (b) from tumour L1, blue region; (c) from tumour L1, green region; (d) from tumour L1, brown region. Fig. S4. Spearman correlation between imaging and histologic, transcriptomics and genomic continuous variables. Fig. S5. Enriched pathways in PC1 and PC2. (a) Principal component 1 (PC1) was found to be significantly associated with the enrichment of pathways involved in lysosomal and intracellular acidification, proteolysis, apoptosis, organic anion transporters, and glutamate and glutamine metabolism. (b) Principal component 2 (PC2) was found to be significantly associated with the enrichment of pathways involved in involved in cellular proliferation, active gene e [file 41747_2025_648_MOESM1_ESM.pdf]

Intra- and inter-tumoral heterogeneity in von Hippel-Lindau disease-related renal cancer: a multimodal data study protocol

ELECTRONIC SUPPLEMENTARY MATERIAL

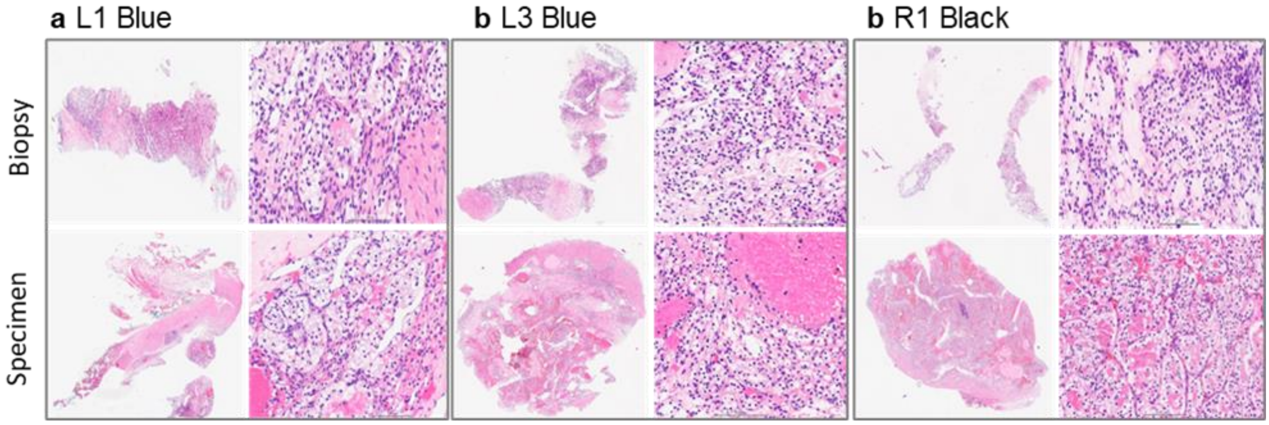

Supplementary Fig. S1. Histology assessment of biopsies and surgical specimen and patient-derived organoid generation. Representative example of histological evaluation of one of the multi-regional biopsies and their corresponding surgical specimens. (**a**) L1 Blue biopsy (upper panel) and corresponding surgical section (lower panel); (**b**) L3 Blue biopsy (upper panel) and corresponding surgical section (lower panel); (**c**) R1 Black biopsy (upper panel) and corresponding surgical section (lower panel).

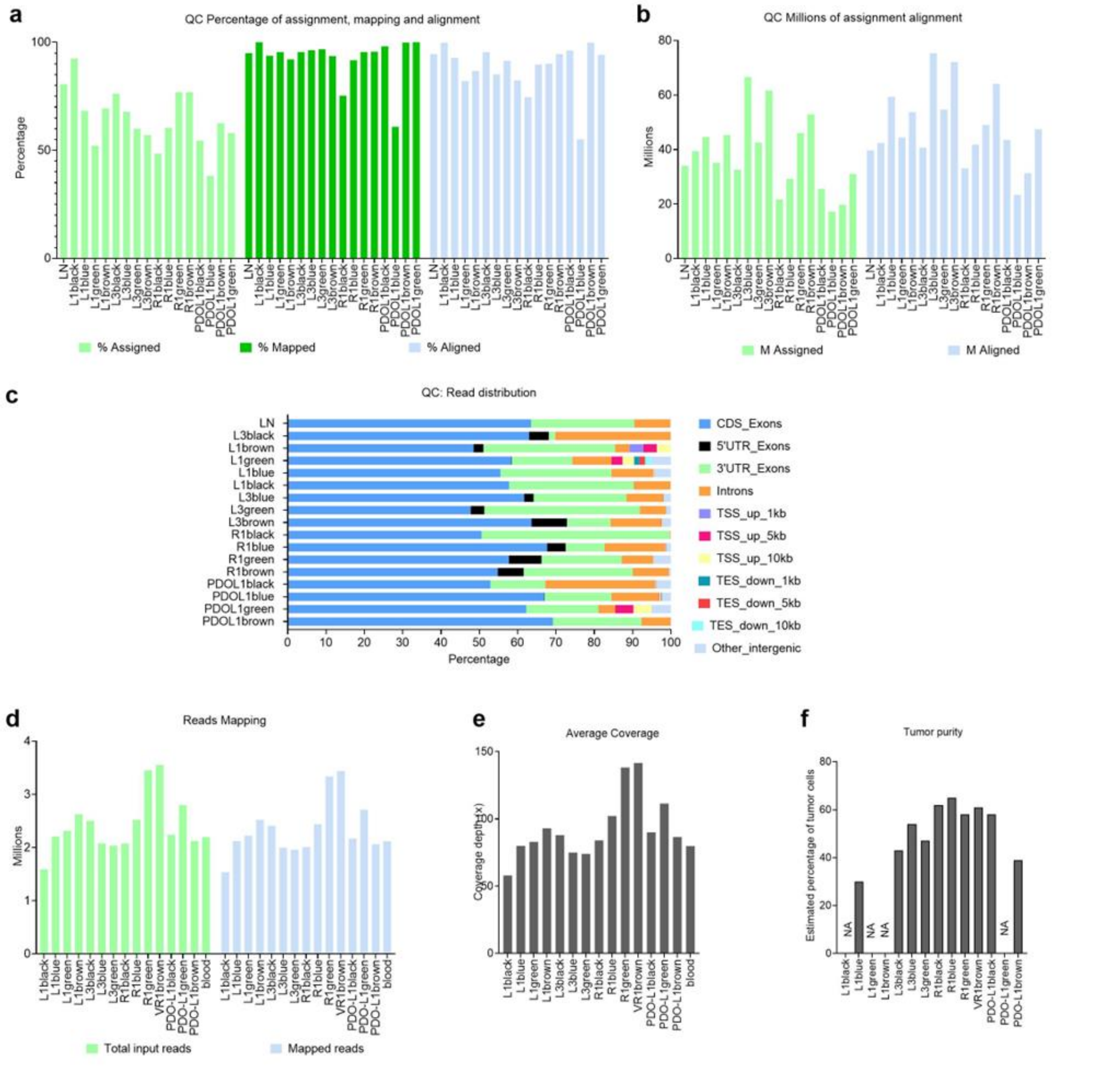

Supplementary Fig. S2. Quality control of RNA and DNA sequencing. Quality control (QC) of RNA sequence: (**a**) percentage of assignment, mapping and alignment; (**b**) millions of assignments and alignment; (**c**) read distribution in each sample. (**d**) Mapping metrics. Total number of input reads and mapped reads for each sample. (**e**) The average coverage over genome was above the recommended target for short-read whole genome sequencing using Illumina platforms (≥ 60× tumour and ≥ 30× blood). (**f**) Estimated tumour purity based on Dynamic Read Analysis for GENomics−DRAGEN somatic pipeline output. *NA* Not available (assigned to samples where the algorithm could not estimate a final tumour model).

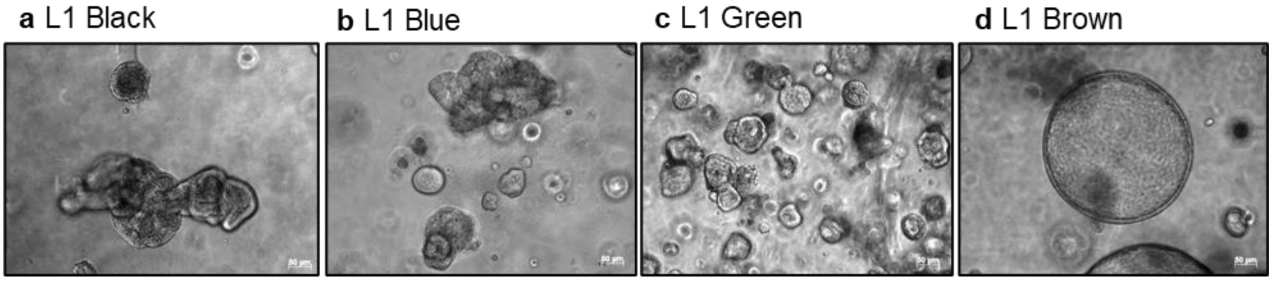

Supplementary Fig. S3. Patient-derived organoids (PDOs) generation. (**a-d**) PDOs cultures from four different tumour regions: (**a**) from tumour L1 black region; (**b**) from tumour L1, blue region; (**c**) from tumour L1, green region; (**d**) from tumour L1, brown region.

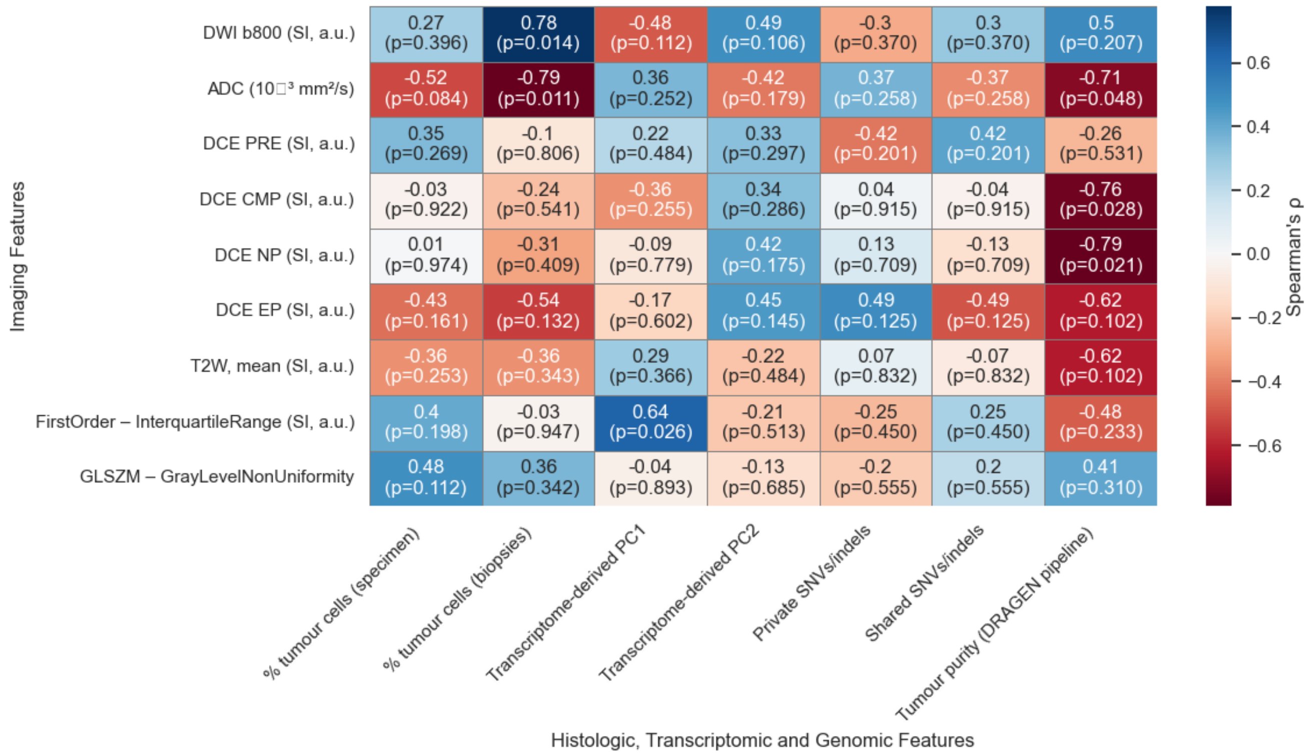

Supplementary Fig. S4. Spearman correlation between imaging and histologic, transcriptomics and genomic continuous variables.

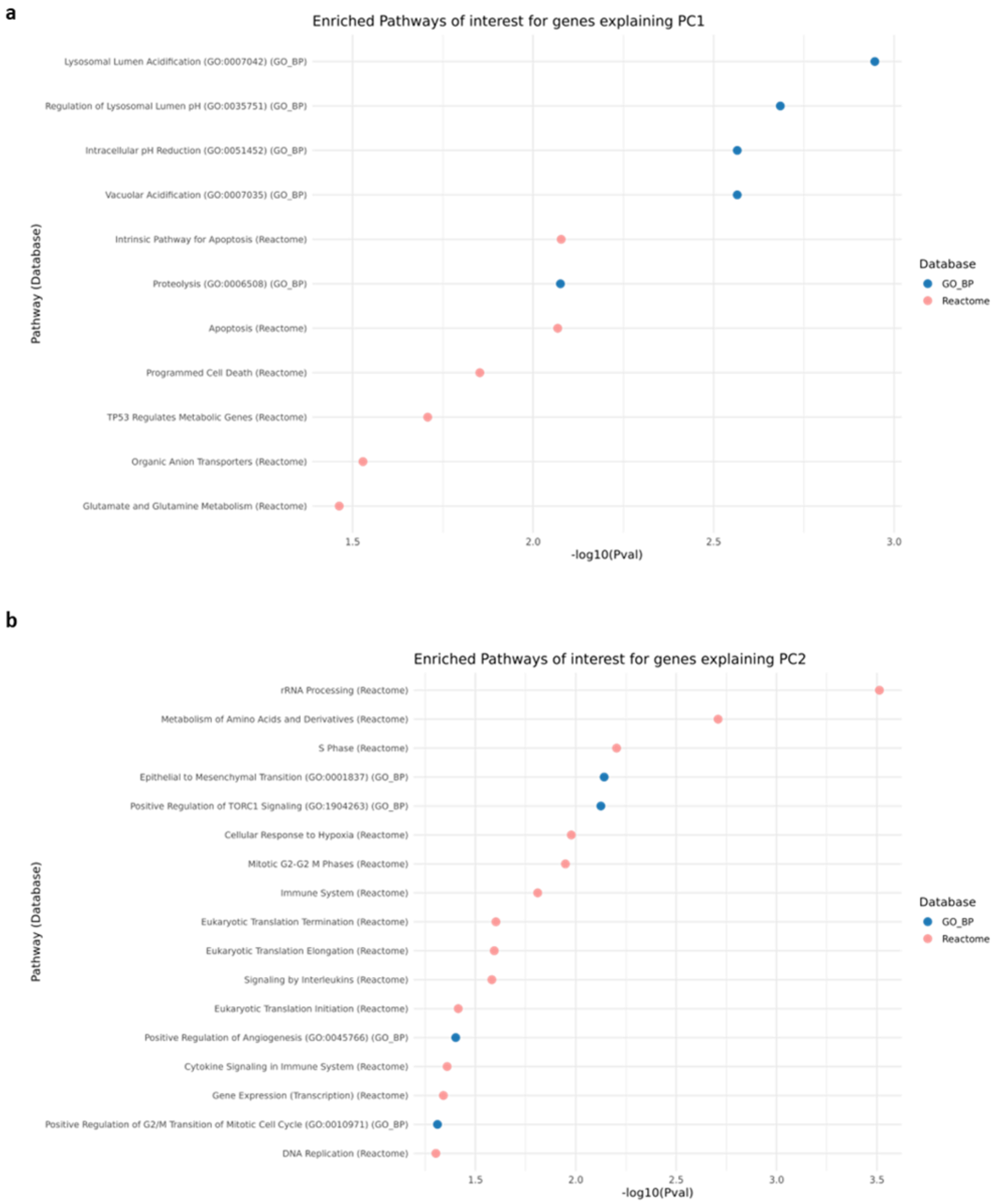

Supplementary Fig. S5. Enriched pathways in PC1 and PC2. (**a**) Principal component 1 (PC1) was found to be significantly associated with the enrichment of pathways involved in lysosomal and intracellular acidification, proteolysis, apoptosis, organic anion transporters, and glutamate and glutamine metabolism. (**b**) Principal component 2 (PC2) was found to be significantly associated with the enrichment of pathways involved in involved in cellular proliferation, active gene expression and translation, epithelial-to-mesenchymal transition (EMT), angiogenesis, hypoxia response, immune activation, and aminoacid metabolism.

Supplementary Table S1. Magnetic resonance imaging acquisition parameters

Parameter	T2-weighted (T2W)	Diffusion-weighted (DWI)	Dynamic contrast enhancement (DCE)
Scanner	1.5-T scanner (Ingenia Ambition S, Philips Medical Systems, Best, the Netherlands)		
Coil	32-channel phased-array coil		
Slice thickness	3 mm	5 mm	5 mm
Slice gap	1 mm	0.6 mm	2.5 mm
Repetition time	2,053 ms	1,218 ms	5.5 ms
Echo time	100 ms	65 ms	1.8/3.8 ms
Field of view	325 × 325 mm^2	375 × 375 mm^2	365 × 365 mm^2
Matrix size	560 × 560 pixels	224 × 224 pixels	400 × 400 pixels
Pixel spacing	0.58 × 0.58 mm	1.67 × 1.67 mm	0.91 × 0.91 mm
Bandwidth	344 Hz/pixel	2,230 Hz/pixel	722 Hz/pixel
b-values	--	0, 400, 800 s/mm^2	--
Apparent Diffusion Coefficient (ADC)	--	Monoexponential fitting	--
Contrast agent	No	No	Gadobutrol (Bayer Healthcare AG, Berlin; Germany); intravenous injection, 0.1 mmol/kg
Phases	--	--	Precontrast (0 s), corticomedullary (25 s), nephrographic (80 s), excretory (300 s)

Supplementary Table S2. Radiomics features maps extraction parameters

Software package	Pyradiomics (v 3.1.0)
Image	T2-weighted (original)
Masks	Whole lesion masks
Preprocessing	Resampled voxel spacing (mm):<br>1 × 1 on the axial plane, depth unchanged
	Image interpolation method: B-spline
	Region-of-interest interpolation method: Nearest neighbour
	Fixed bin width: 25
Feature extraction	Voxel-based
	Force2D: True
	Force2DDimension: 0 (axial plane)
Feature classes	First Order Statistics<br>Gray Level Size Zone Matrix (GLSZM)<br>Neighboring Gray Tone Difference Matrix (NGTDM)<br>Gray Level Dependece Matrix (GLDM)

Supplementary Table S3. Nested ANOVA results – Multiparametric analysis

Nested ANOVA						
Variable	F inter	F Intra	*p*-value inter	*p*-value intra	η^2 Inter*	η^2 Intra*
DWI b800	359.829	119.768	< 0.0001	< 0.0001	0.119	0.178
ADC	892.080	161.126	< 0.0001	< 0.0001	0.238	0.194
DCE PRE	1,109.634	64.896	< 0.0001	< 0.0001	0.315	0.083
DCE CMP	90.247	110.279	< 0.0001	< 0.0001	0.033	0.183
DCE NP	57.386	46.881	< 0.0001	< 0.0001	0.024	0.088
DCE EP	9.012	47.513	0.0001	< 0.0001	0.004	0.091
Inter-tumor comparisons						
Variable	Var 1	Var 2	Mean ± SD (1)	Mean ± SD (2)	η^2 **	*p*-value
DWI b800	R1	L1	239.52 ± 48.61	204.60 ± 19.58	0.444	< 0.0001
DWI b800	R1	L3	239.52 ± 48.61	215.59 ± 41.07	0.141	< 0.0001
DWI b800	L3	L1	215.59 ± 41.07	204.60 ± 19.58	0.058	< 0.0001
ADC	R1	L1	1.83 ± 0.46	2.27 ± 0.15	0.825	< 0.0001
ADC	R1	L3	1.83 ± 0.46	2.14 ± 0.24	0.351	< 0.0001
ADC	L3	L1	2.14 ± 0.24	2.27 ± 0.15	0.218	< 0.0001
DCE PRE	R1	L3	426.57 ± 63.70	570.12 ± 143.25	0.838	< 0.0001
DCE PRE	L3	L1	570.12 ± 143.25	435.97 ± 63.18	0.734	< 0.0001
DCE PRE	R1	L1	426.57 ± 63.70	435.97 ± 63.18	0.011	0.0003
DCE NP	L3	L1	1904.65 ± 400.88	1721.47 ± 529.05	0.076	< 0.0001
DCE NP	R1	L1	1831.44 ± 419.73	1721.47 ± 529.05	0.027	< 0.0001
DCE NP	R1	L3	1831.44 ± 419.73	1904.65 ± 400.88	0.016	< 0.0001
DCE EP	R1	L1	1896.06 ± 477.49	1962.81 ± 491.88	0.009	0.0009
DCE EP	R1	L3	1896.06 ± 477.49	1945.81 ± 432.02	0.006	0.0076
DCE EP	L3	L1	1945.81 ± 432.02	1962.81 ± 491.88	0.001	1.0000
DCE CMP	L3	L1	1601.96 ± 333.70	1405.87 ± 487.84	0.110	< 0.0001
DCE CMP	R1	L1	1549.89 ± 417.28	1405.87 ± 487.84	0.050	< 0.0001
DCE CMP	R1	L3	1549.89 ± 417.28	1601.96 ± 333.70	0.009	0.0005
Nested ANOVA post-hoc tests (15 highest η2)						
Variable	Lesion	Quadrants	Mean ± SD (1)	Mean ± SD (2)	η^2 **	P-Value
DCE PRE	L1	Black - Green	409.99 ± 54.72	501.49 ± 46.84	1.6136	< 0.0001
DWI b800	L3	Green - Black	243.44 ± 30.89	182.43 ± 38.78	1.5147	< 0.0001
ADC	L1	Black - Green	2.35 ± 0.11	2.17 ± 0.11	1.3389	< 0.0001
ADC	R1	Brown - Blue	2.23 ± 0.18	1.70 ± 0.43	1.2466	< 0.0001
ADC	L3	Brown - Black	2.04 ± 0.15	2.32 ± 0.20	1.229	< 0.0001
DCE PRE	L1	Blue - Green	407.99 ± 72.13	501.49 ± 46.84	1.1819	< 0.0001
ADC	R1	Brown - Black	2.23 ± 0.18	1.51 ± 0.65	1.1104	< 0.0001
DCE CMP	L1	Black - Blue	1206.83 ± 337.47	1785.40 ± 512.69	0.8885	< 0.0001
DCE CMP	L1	Blue - Brown	1785.40 ± 512.69	1213.07 ± 354.38	0.8433	< 0.0001
ADC	L3	Green - Black	2.02 ± 0.26	2.32 ± 0.20	0.8108	< 0.0001
DWI b800	R1	Brown - Blue	201.09 ± 36.94	259.43 ± 53.41	0.8069	< 0.0001
DWI b800	L3	Brown - Black	228.12 ± 35.98	182.43 ± 38.78	0.7463	< 0.0001

DCE CMP	L1	Black - Green	1206.83 ± 337.47	1725.11 ± 501.08	0.736	< 0.0001
ADC	R1	Green - Brown	2.03 ± 0.15	2.23 ± 0.18	0.7001	< 0.0001
DCE CMP	L1	Brown - Green	1213.07 ± 354.38	1725.11 ± 501.08	0.6961	< 0.0001

* η^2 effect size defined as between/within-group variance divided by total variance

** η^2 effect size defined as standardized mean differences

ADC Apparent diffusion coefficient, *CMP* Cortico-medullary phase, *DCE* Dynamic contrast enhancement, *DWI* Diffusion-weighted imaging, *EP* Excretory phase, *NP* Nephrogenic phase, *PRE* Precontrast, *SD* Standard deviation.

Supplementary Table S4 Nested ANOVA results – Radiomics

Nested ANOVA

Variable	F inter	F Intra	*p*-value inter	*p*-value intra	η^2 inter*	η^2 intra*
firstorder_Entropy	3.462	3.194	0.0316	0.0008	0.005	0.019
firstorder_InterquartileRange	34.483	10.861	< 0.0001	< 0.0001	0.042	0.060
firstorder_Kurtosis	5.903	2.344	0.0028	0.0127	0.008	0.014
firstorder_Skewness	3.699	1.777	0.0250	0.0681	0.005	0.011
glszm_GrayLevelNonUniformity	7.304	1.549	0.0007	0.1257	0.010	0.009
ngtdm_Busyness	2.013	6.085	0.1339	< 0.0001	0.003	0.036
T2W	225.4	19.410	< 0.0001	< 0.0001	0.216	0.084

Inter-tumor comparisons

Variable	Var 1	Var 2	Mean ± SD (1)	Mean ± SD (2)	η^2 **	*p*-value
firstorder_Entropy	L3	L1	2.58 ± 0.37	2.52 ± 0.37	0.015	0.0389
firstorder_Entropy	R1	L1	2.57 ± 0.36	2.52 ± 0.37	0.009	0.1229
firstorder_Entropy	R1	L3	2.57 ± 0.36	2.58 ± 0.37	0.001	1.0000
firstorder_InterquartileRange	R1	L3	131.87 ± 82.78	175.72 ± 118.13	0.092	< 0.0001
firstorder_InterquartileRange	L3	L1	175.72 ± 118.13	136.48 ± 74.39	0.079	< 0.0001
firstorder_InterquartileRange	R1	L1	131.87 ± 82.78	136.48 ± 74.39	0.002	1.0000
firstorder_Kurtosis	R1	L3	2.25 ± 0.68	2.12 ± 0.62	0.018	0.0063
firstorder_Kurtosis	R1	L1	2.25 ± 0.68	2.14 ± 0.61	0.013	0.0392
firstorder_Kurtosis	L3	L1	2.12 ± 0.62	2.14 ± 0.61	0.000	1.0000
firstorder_Skewness	R1	L1	-0.04 ± 0.58	-0.13 ± 0.53	0.015	0.0279
firstorder_Skewness	R1	L3	-0.04 ± 0.58	-0.10 ± 0.57	0.006	0.2416
firstorder_Skewness	L3	L1	-0.10 ± 0.57	-0.13 ± 0.53	0.002	1.0000
glszm_GrayLevelNonUniformity	R1	L3	1.13 ± 0.17	1.10 ± 0.16	0.022	0.0020
glszm_GrayLevelNonUniformity	R1	L1	1.13 ± 0.17	1.10 ± 0.18	0.018	0.0091
glszm_GrayLevelNonUniformity	L3	L1	1.10 ± 0.16	1.10 ± 0.18	0.000	1.0000
ngtdm_Busyness	R1	L1	0.08 ± 0.06	0.08 ± 0.07	0.007	0.1811
ngtdm_Busyness	L3	L1	0.07 ± 0.08	0.08 ± 0.07	0.006	0.3100
ngtdm_Busyness	R1	L3	0.08 ± 0.06	0.07 ± 0.08	0.000	1.0000
T2W	R1	L1	1295.60 ± 214.87	1559.12 ± 215.24	0.751	< 0.0001
T2W	R1	L3	1295.60 ± 214.87	1517.66 ± 253.75	0.446	< 0.0001
T2W	L3	L1	1517.66 ± 253.75	1559.12 ± 215.24	0.016	0.0329

Nested ANOVA post-hoc tests (15 highest η^2)

Variable	Lesion	Quadrants	Mean ± SD (1)	Mean ± SD (2)	η^2 **	*p*-Value
T2W	L1	Black - Green	1658.86 ± 187.49	1380.04 ± 175.04	1.1816	< 0.0001
T2W	L1	Brown - Green	1622.75 ± 172.36	1380.04 ± 175.04	0.9761	< 0.0001
T2W	L1	Black - Blue	1658.86 ± 187.49	1451.00 ± 207.40	0.5527	< 0.0001
firstorder_InterquartileRange	L3	Green - Brown	129.43 ± 76.02	235.48 ± 141.94	0.4338	< 0.0001
T2W	L3	Blue - Green	1589.08 ± 203.80	1422.23 ± 164.41	0.4060	< 0.0001
T2W	L1	Blue - Brown	1451.00 ± 207.40	1622.75 ± 172.36	0.4056	< 0.0001
T2W	L3	Green - Black	1422.23 ± 164.41	1587.15 ± 293.16	0.2408	< 0.0001
firstorder_InterquartileRange	L3	Green - Black	129.43 ± 76.02	186.87 ± 105.65	0.1948	< 0.0001
firstorder_InterquartileRange	L3	Blue - Brown	155.47 ± 122.74	235.48 ± 141.94	0.1818	0.0001
ngtdm_Busyness	L1	Black - Blue	0.07 ± 0.04	0.12 ± 0.11	0.1804	< 0.0001

T2W	L3	Blue - Brown	1589.08 ± 203.80	1448.37 ± 273.03	0.1706	0.0002
ngtdm_Busyness	L1	Black - Green	0.07 ± 0.04	0.10 ± 0.08	0.1675	0.0002
ngtdm_Busyness	L1	Blue - Brown	0.12 ± 0.11	0.07 ± 0.05	0.1480	0.0005
ngtdm_Busyness	R1	Brown - Black	0.10 ± 0.10	0.06 ± 0.05	0.1371	0.0024
ngtdm_Busyness	L1	Brown - Green	0.07 ± 0.05	0.10 ± 0.08	0.1256	0.0037

* η^2 effect size defined as between/within-group variance divided by total variance.

** η^2 effect size defined as standardized mean differences.

T2W T2-weighted Imaging, GLSZM Gray Level Size Zone Matrix, *NGTDM* Neighboring Gray Tone Difference Matrix, *GLDM* Gray Level Dependence Matrix.

Supplementary methods

Sampling of multiregional biopsies

The excised tumour was divided into four equal sections and core 16-gauge needle biopsies were taken from each quadrant.

Histology of multiregional biopsies and surgical sections

Histopathological assessment was made by pathologists with dedicated experience in the diagnosis of RCC. Biopsies, and the corresponding surgical sections were cut and stained for Haematoxylin and Eosin using the Ventana HE 600 system. Designated pathologists assessed histology, stage, grading WHO/ISUP (G1-G4), necrosis, angioinvasion, and tumour cell percentage.

Simultaneous purification of genomic DNA and total RNA from multiregional tumour biopsies

Genomic DNA and total RNA were simultaneously purified from biopsies of 1.2 mm of diameter and 5-10 mm in length. We used QIAGEN AllPrep DNA/RNA Micro kit according to the manufacturer’s instructions, using a glass/Teflon potter in nitrogen to pulverize the samples. The lysates were kept overnight at -80°C and homogenized with a 20-gauge needle. Nucleic acids integrity was assessed with TapeStation (Agilent technologies, Santa Clara, USA). The concentration of the DNA samples was assessed with the Qubit fluorimeter (Thermo Fisher Scientific, Waltham, U.S.A).

RNA sequencing and transcriptomic data analysis

Sequencing libraries were prepared using the "Low_Input_mRNA_Novaseq6000_Gb" protocol, targeting 1x100nt reads, and sequenced on the NovaSeq 6000 platform (Illumina, San Diego, USA). The quality of the raw sequencing reads was assessed using FastQC version 0.11.8 (Andrews, 2010), and quality control reports were subsequently aggregated with MultiQC version 1.9 (Ewels et al., 2016). Adapter sequences were removed from the raw reads using Cutadapt version 2.7 (Martin, 2011) and reads were aligned to the human reference genome (GRCh38) using STAR version 2.5.3a (Dobin et al., 2013). The alignments were based on the Gencode Release 44 gene annotation and Ensembl Release 110.

Gene expression was quantified using FeatureCounts version 1.6.4 (Liao et al., 2014). The raw count data matrix was imported into R environment (R version 4.3.2) and processed using the DESeq2 vs 1.42.1 workflow (Love et al., 2014), which includes normalization of the data and identification of differentially expressed genes based on a negative binomial distribution model.

A first Principal Component Analysis (PCA) was performed to assess the overall variation in gene expression across the tumours. PCA was performed for the three pairwise comparisons. The *Variance Stabilizing Transformation (vst)* function from DESeq2 was applied to the count data to stabilize the variance across genes before performing PCA. The PCA plot was generated using the top 500 most variable genes, as identified by vst, to visualize clustering and overall sample distribution.

A distinct PCA analysis was performed to evaluate gene expression variation across all the tumour regions. The proportion of variance explained by each principal component was calculated, and the first two PCs were retained for subsequent analyses (each explaining more than 10% of the variance). For PCs showing meaningful correlations with radiomic features, loadings were extracted to identify the top 50 contributing genes. These gene lists were used for functional enrichment analysis using the EnrichR R package, considering the following databases: · GO_Biological_Process_2025 and Reactome_Pathways_2024.

Patient-derived organoid cultures (PDOs)

Tumour biopsies were cut into 2–4 mm pieces and dissociated into single-cell suspensions using the Tumour Dissociation Kit (Miltenyi Biotec, Bergisch Gladbach, Germany) and gentleMACS™ Dissociators with heaters, following the manufacturer’s protocol. The samples were filtered through a 70 µm cell strainer, treated with erythrocyte lysis buffer for 5 min at room temperature, and centrifuged at 300 × g for 5 min. Cell pellets were resuspended in 75% Matrigel (Corning, CLS356231) diluted in growth medium (DMEM F-12 supplemented with 1x B27, 20 ng/ml EGF, 20 ng/ml FGFb, 4 µg/ml heparin, 10 mM Hepes, 10 µM Y27632, 5 µM A83-01, 1x Pen/Strep, 125 µg/ml Amphotericin B) and seeded as 50 µl droplets in 96-well plates. Plates were inverted for 15 min at 37°C to solidify Matrigel, after which 150 µl of growth medium was added and refreshed every 3–4 days. PDOs were passaged upon reaching diameters of 300–500 µm.

Additional references

1. Andrews, S. (2010). FastQC: A Quality Control tool for High Throughput Sequence Data. Available online: https://www.bioinformatics.babraham.ac.uk/projects/fastqc/
2. Ewels, P., Magnusson, M., Lundin, S., & Käller, M. (2016). "MultiQC: summarize analysis results for multiple tools." *Bioinformatics*, 32(19), 3047-3048. https://doi.org/10.1093/bioinformatics/btw354
3. Martin, M. (2011). "Cutadapt removes adapter sequences from high-throughput sequencing reads." *EMBnet.journal*, 17(1), 10-12. https://doi.org/10.14806/ej.17.1.200
4. Dobin, A., Davis, C. A., Schlesinger, F., et al. (2013). "STAR: ultrafast universal RNA-seq aligner." *Bioinformatics*, 29(1), 15-21. https://doi.org/10.1093/bioinformatics/bts635
5. Liao, Y., Smyth, G. K., & Shi, W. (2014). "featureCounts: an efficient general purpose program for assigning sequence reads to genomic features." *Bioinformatics*, 30(7), 923-930. https://doi.org/10.1093/bioinformatics/btt656
6. Love, M. I., Huber, W., & Anders, S. (2014). "Moderated estimation of fold change and dispersion for RNA-seq data with DESeq2." *Genome Biology*, 15(12), 550. https://doi.org/10.1186/s13059-014-0550-8
